# Made to measure—Selecting outcomes in aneurysmal subarachnoid hemorrhage research

**DOI:** 10.3389/fneur.2022.1000454

**Published:** 2022-09-21

**Authors:** Christopher R. Andersen, Shane W. English, Anthony Delaney

**Affiliations:** ^1^Northern Clinical School, Sydney University, Sydney, NSW, Australia; ^2^The George Institute for Global Health, UNSW, Sydney, NSW, Australia; ^3^Intensive Care Department, Royal North Shore Hospital, Sydney, NSW, Australia; ^4^Department of Medicine (Critical Care), uOttawa, Ottawa, ON, Canada; ^5^Ottawa Hospital Research Institute (OHRI), Ottawa, ON, Canada

**Keywords:** subarachnoid hemorrhage, outcome assessment (health care), core outcome set (COS), measurement instruments, patient reported outcome measures, stroke

## Abstract

There has been limited new high-level evidence generated to guide aneurysmal subarachnoid hemorrhage (aSAH) management in the past decade. The choice of outcome measures used in aSAH clinical trials may be one of the factors hindering progress. In this narrative review we consider the current process for determining “what” to measure in aSAH and identify some of the shortcomings of these approaches. A consideration of the unique clinical course of aSAH is then discussed and how this impacts on selecting the best timepoints to assess change in the chosen constructs. We also review the how to critically appraise different measurement instruments and some of the issues with how these are applied in the context of aSAH. We conclude with current initiatives to improve outcome selection in aSAH and future directions in the research agenda.

## Introduction

Aneurysmal subarachnoid hemorrhage (aSAH) is a devastating type of stroke that is caused by the rupture of an abnormal intracranial artery. It is associated with a high degree of mortality and a majority of survivors are left with long-term morbidity (1, 2). Despite the burden this condition places on patients, their families and society more generally, there is limited high-level evidence to guide treatment (3, 4). As such aSAH is, and will remain, an area of significant research interest. Ensuring that this research is efficient and well-designed to meet the needs of patients and health care providers is crucial.

One area of research design that is often overlooked is the selection of outcome measures. Getting the outcome measure selection right is fundamental for ensuring that the inferences that we draw from clinical research are valid and patient focused. Using a poorly chosen primary outcome measure might miss a clinical important benefit, or a surrogate outcome may suggest benefit of an intervention when there is none, or worse harm. Research is littered with examples of both (5, 6).

**Figure 1:**
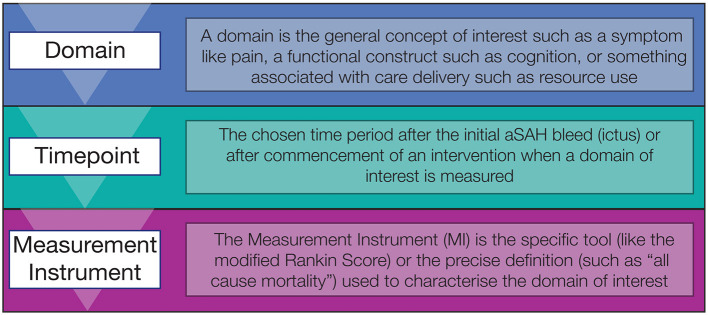
Three step process for selecting outcome measures in aSAH, starting with the domain of interest, the timepoint and then measurement instrument.

Choice of the outcome domain of interest by researchers is guided by the stage of research, population of interest, intervention being studied, and the comparator chosen. Researchers should consider the domains or outcomes that are most relevant to the research question and when is the most relevant timepoint/s for assessing this outcome (see Figure 1). They should then review the available instruments to determine which is best able to detect clinically meaningful change in this domain (7). Central to this process should be the end users of clinical research, patients and caregivers, policy makers, and the health care professionals that provide care.

## What to measure: Choosing the right domains

**Figure 2:**
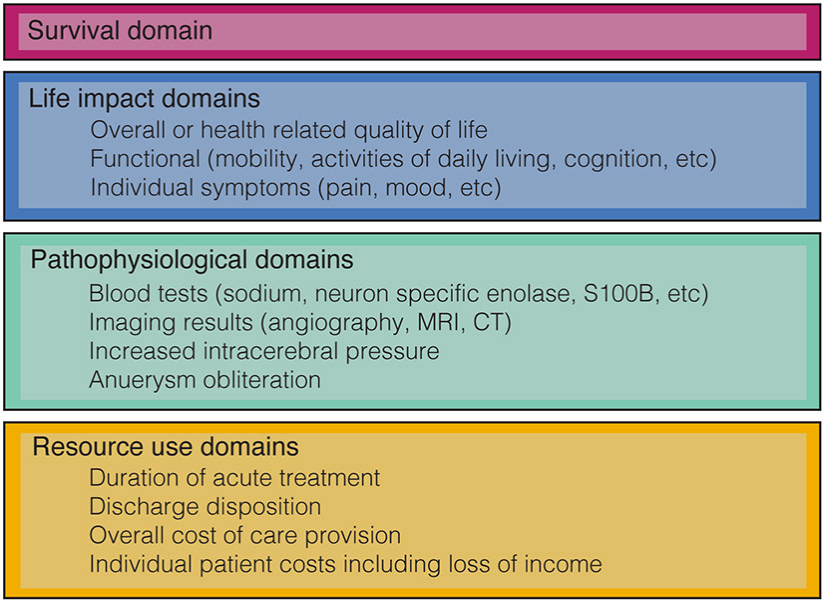
Classification of some of the common outcome domains used in aSAH research based on the OMERACT filter 2.0 (10).

Outcome domains are defined as the aspects of a medical condition that are important to patients, researchers, and health care providers and may be used to measure or assess the effects of interventions. Conceptually, outcome domains can be considered across a continuum of increasing complexity from pathophysiological variables such as blood tests or imaging results, through individual symptoms, then assessments of body function or activities, to patient perceptions of health and overall measures of quality of life (QoL) (8). Other commonly used taxonomies such as those developed by the OMERACT investigators also include domains such as survival and resource use (see Figure 2) (9, 10). As outcome domains progress along the continuum from pathophysiological through to overall measures of QoL they generally increase in authority and their ability influence clinical practice.

In aSAH, the different outcome domains chosen by researchers have varied significantly and been inconsistent across studies. In a review of 129 randomized controlled trials (RCTs) over a 20 year period survival is the most commonly reported outcome domain although it is only used as a primary outcome in 4% of studies (11). A measure of general patient function is also reported in more than half of the included studies, one in five used a functional measure as the primary outcome. Measures of how a patient feels, functions, or survives were used as a primary outcome in less than half of the studies, although those with higher numbers of participants were more likely to choose clinically meaningful outcome measures. Surrogate outcomes such as radiological vasospasm or transcranial doppler findings are much more likely to be reported and used as a primary outcome than measures of cognition or QoL. Patient reported QoL was only reported in 8.5% of included studies and its use as a primary outcome was only reported in one study.

Although pathophysiological domains are often easier to measure and demonstrate a statistically significant difference they should be interpreted with caution. Radiological vasospasm provides an important insight to this in aSAH research. After mortality and functional outcomes, angiographic vasospasm (as demonstrated with an imaging modality such as transcranial doppler or angiography) is the third most reported domain in aSAH RCTs with 8% of studies also choosing this as a primary outcome measure (11). Angiographic vasospasm seems a reasonable candidate for a pathophysiological outcome domain. It has both a biologically plausible mechanism for causing cerebral infarction and there is a strong association between the presence of angiographic vasospasm and poor outcomes. Notably however, the results of the well-designed CONSCIOUS-1 and CONSCIOUS-2 studies showed the use of clazosentan resulted in a dose-dependent reduction in angiographic vasospasm but did not show an improvement in clinically meaningful outcomes (12–14). Angiographic vasospasm therefore has biological plausibility and a strong association, but the best available evidence does not demonstrate that reducing vasospasm leads to an improvement in a more complex outcome describing how a patient feels, functions, or survives and therefore it should be used in research with caution (15, 16).

Another potential issue in aSAH research is the selection of outcome domains by researchers that do not meet the needs of the main end users of the research: patients and health care professionals. Multiple studies in the medical literature have shown that there is often a mismatch between the domains selected by researchers and those that are prioritized by end users (5, 17, 18). A recent systematic review did not identify any articles that described what domains aSAH patient or family prioritize in clinical research (19). It did however identify six categories of outcome domains that respondents felt helped or hindered their recovery including cognitive, physical, emotional, psychological, social, and emotional domains. Most of these domains are not well–reported in aSAH RCTs (11). The lack of understanding with respect to what outcome domains key stakeholder prioritize and likely mismatch remains an ongoing issue in aSAH research.

## When to measure: Choosing the right timepoints

When the specific domains of interest have been established, it is important to determine the optimal time point for measurement. Ideally, researchers should be guided by temporal analysis of the domain of interest, estimating the earliest time point where there is minimal further change attributable to the intervention being evaluated. Decisions regarding optimal timepoints will also be influenced by the logistical challenges of conducting trial research. The longer the interval for assessment the higher the risks of loss to follow up and generally the higher the costs of conducting the research. There is also a concern that potentially beneficial treatments are delayed from being available if the time to outcome assessment is extended.

Unlike many remitting-relapsing conditions such as rheumatoid arthritis, aSAH follows a characteristic time course from ictus. This makes the decision of timepoints also relevant in terms of the disease trajectory as well as the timing of the intervention. Patients often have a hyper-acute phase of early brain injury in the first 24–48 h followed delayed brain injury in the subsequent weeks due to complications such as delayed cerebral ischemia, hospital acquired infection, hydrocephalus and rebleeding (20, 21). In the weeks after ictus some pathophysiological outcome domains such as cerebral infarction will become settled with minimal further change expected in the subsequent months (22). Some outcome domains such as resource use may be determined at landmark events such as intensive care or hospital discharge. More complex outcomes such as function and QoL measures generally remain dynamic for months after the initial ictus. The researchers of the landmark International Subarachnoid Aneurysm Trial used a functional domain (including survival) at 12 months from ictus as the primary outcome, but as there was potential change attributable the interventions over a longer period of time, continued to report timepoints years after the intervention in subsequent publications (23, 24).

Current selection of timepoints in aSAH RCTs is highly variable even within commonly reported domains. Within functional domains, the two most reported scores are reported at as early as 10 days post ictus up to many years post ictus. The most common timepoint for functional domains and survival is 3 months, with 6 and 12 months also frequently reported (11). This variability can make comparing different studies more challenging and may lead to research waste.

## How to measure: Choosing the right measurement instruments

Once the domain and timepoints have been determined, researchers need to find the best measurement instrument (MI) to operationalize the construct of interest. An ideal MI should be reliable, valid, responsive and interpretable (25). A reliable MI should give consistent results every time it is used. It should have minimal sources of variation beyond the intervention of interest and should attempt to estimate these sources of variation (the measurement error) so they can be standardized (26). Validity relates to the MI accurately measuring the construct of interest. Responsiveness is the ability of the MI to detect a change in the domain of interest. Interpretability of a MI such as mortality at 3 months is straightforward but this may not necessarily be the case with more complex MI such as a patient reported QoL scale. A patient reported questionnaire will need to ensure users understand how the scores are distributed and what a minimally important clinical difference might be to address interpretability.

There is a wide range of pathophysiological domains that are reported in aSAH with radiological vasospasm, cerebral infarction and delayed cerebral ischemia the most commonly reported in RCTs (11). The MIs and definitions for these domains are however highly variable. With respect to radiological vasospasm, studies have used transcranial doppler of the middle cerebral artery and compared mean thresholds or a Lindegaard ratio (27) or alternatively have used digital subtraction angiography, or CT angiography (28–30). Whilst there are advantages and disadvantages to each of these methods it is unclear which is the best way to measure radiological vasospasm. DCI has also been defined in many ways including delayed neurological deterioration, symptomatic vasospasm and clinical vasospasm (31–33). This heterogeneity limits our ability to interpret and draw inferences with respect to the true impact of an intervention and compare results between studies (34).

Measuring resource use is usually done in terms of time increments (duration of hospital admission, length of therapy) or in financial cost. Some of this data such as length of stay or number of procedures are more easily recorded or able to be extracted from administrative data including the electronic patient record. Length of hospital stay is the most commonly reported resource use measure in aSAH but non–clinical factors such as insurance status and local institutional policies introduce significant variability that may reduce reliability (11, 35). Measuring the time spent at home in the 90 days post ictus is a novel measure of disposition that may also be a reasonable surrogate for functional outcomes (36). Combining administrative information with patient-reported questionnaires could identify more nuanced data relevant to patients such as reduction in income and other costs that are more difficult to measure and may improve validity by providing a more accurate measure of resource use (37).

Function can be conceptualized through specific body functions (such as cognition or dexterity), through activities (such as mobility and self-care) and participation (such as engagement in work or social life) (38). Despite a pattern of neurological sequelae that is distinct to other forms of neurological conditions there are no functional MI that have been developed specifically for aSAH in common use. The modified Rankin score (mRS) and the Glasgow Outcome Scale (GOS) were developed for stroke patients and brain injury patients respectively (39–41). These functional scores assess activity and are frequently reported and used as a primary outcome in aSAH research. Assessment specific neurological functioning such as the Finger Tapping Test (dexterity), Verbal Fluency Test (language), Trail Marking Test (executive function) and Weschler Adult Intelligence Scale (cognition) have been used in only limited settings (42–45).

The modified Rankin score (mRS) is an ordinal scale of increasing disability that was developed for use in stroke patients. In recent times the mRS has become the most used functional outcome MI in aSAH research (11). Whilst the validity and reliability of the mRS is well–studied in stroke patients it's measurement properties with respect to aSAH are less well–studied (46, 47). There is evidence that the method of mRS acquisition in aSAH could introduce variance and therefore reduce the reliability (48). It is also somewhat limited and many important body function characteristics such as cognition are not captured. This limitation implies a ceiling to the mRS, whereby many patients that may have a good assessment based on self-care but still have significant impairment related to other body functions is not captured (49, 50). In large international trials the mRS as a primary endpoint has demonstrated a clinical important difference in functional outcome although a more comprehensive scale without the associated ceiling effects may be more responsive and allow smaller sample sizes (24).

Many functional scales such as the mRS and GOS are dichotomized into “good” and “poor” outcomes which improves its interpretability but how this is done is not consistent (11). Ideally researchers should consider the distribution of mRS outcomes, but this may not be known prior to study commencement. Work by the SAHIT investigators has shown that in some trials up to 75% of patients are classified as a “good” outcome reducing the power of this MI (49). In stroke research there has been a push to use the full ordinal scale when employing the mRS to improve the statistical power and identify change across the whole ordinal range despite the perceived reduction in interpretability (51, 52).

As outcome domains become more complex it becomes increasingly crucial that the MI is assessed from the patient's perspective to ensure that the assessment of health status is accurate (53). Patient reported outcome measures (PROMs) are when a patient or caregiver directly reports symptoms, function or an assessment of quality of life (54, 55). There is currently only limited application of PROMs in aSAH research with these MI reported in < 10% of recent RCTs (11). The PROMs used in recent RCTs are primarily generic measures of QoL including the Short Form 36 Health Survey and the EQ5D Score (56, 57). Some neurological PROMS have been assessed in the context of aSAH patients such as the Stroke-Specific Quality of Life Scale, the Neuro-QoL and Quality of Life after Brain Injury Overall Scale (58–62). More recently there have emerged several aSAH specific PROMs with the subarachnoid hemorrhage specific outcome tool (SAHOT) and the Screening for Symptoms in Aneurysmal Subarachnoid Hemorrhage (SOS-SAH) questionnaire (63, 64). Assessment for a PROMs reliability, validity, responsiveness, and interpretability is challenging and more work needs to be done to further evaluate these instruments. Notably a recent systematic review of PROMs (published prior to the SOS-SAH) concluded that there is currently an insufficient evidence base for selecting an appropriate PROM in aSAH (65).

## Moving forward

Moving forward there are multiple ways in which to improve outcome selection in aSAH research. One of the most important reforms is to ensure that there is patient and care giver involvement through all stages of the research process. This will help address any potential mismatch between the outcomes chosen by researchers and those prioritized by patients and health care providers. Patient involvement in outcomes research has been shown to widen the research agenda and led to the use of more patient relevant outcomes in clinical trials (66).

Pathophysiological outcomes remain important especially in early phase research and pilot studies. It is recommended that researchers move away from targeting pathophysiological outcomes such as radiological vasospasm and use alternative domains such as delayed cerebral ischemia and radiological evidence of cerebral infarction. These domains are likely to offer more robust insights to intervention efficacy (67, 68). Definitive research should continue to only use primary outcomes domains that represent how a patient feels, functions, or survives.

Choosing timepoints that can be standardized in the context of aSAH remains a challenge. Ideally, analysis of large databases and trial data repositories such as the SAHIT will allow better understanding of the trajectories of different outcome domains and help researchers identify ideal timepoints. Key stakeholders including patients and health care providers should also be consulted. In the interim it is recommended that researchers follow current expert consensus to use 3 months after ictus for most outcomes with a clear ceiling effect and longer time periods where there may be ongoing change such as rebleeding or recanalization (69).

Several notable standardization initiatives in aSAH research have improved the selection and use of outcome measures. The work by an international panel of experts over a decade ago proposed standardized definitions for DCI and cerebral infarction and this has helped address variable reporting of these instruments in aSAH research (34). More recently there has been important work by the National Institute of Neurological Diseases and Stroke Common Data Elements (NINDS-CDE) (70). This work classified over 50 MI into core, supplemental-highly recommended, supplemental, and exploratory. It also provided detailed case report forms to improve consistency of reporting. The mRS and the Montreal Cognitive Assessment were highly recommended supplemental MI, with the GOS, GOS-Extended, and Death as supplemental and all other outcomes classified as exploratory. The expert panel did not identify any core outcomes however (69).

When a domain and timepoint has been identified, it is recommended that researchers evaluate potential MI for reliability, validity, responsiveness, and interpretability. The Consensus-based Standards for the Selection of health Measurement INstruments (COSMIN) provides detailed advice and tools that use these criteria to determine the most appropriate MI (71). With respect to PROMs some of this work has been completed and may only need updating to account for the recently developed PROMs (65).

Ideally, MIs that assess domains for symptoms, functional domains and overall quality of life should be developed for specifically for an aSAH cohort with aSAH patient involvement given the unique sequalae and clinical course of the disease. The two recently developed aSAH PROMs are a promising start but require further testing and evaluation (63, 64). There is a need for aSAH specific functional measures that do not have the ceiling effects of current scales and include important domains such as cognition. We are not aware of any new functional measures being developed however and it is likely that mRS and GOS will continue as the most commonly applied functional MI. As such it is recommended that analysis using the full ordinal scale is considered even when this occurs at the cost of some interpretability. If it is decided to progress with dichotomization then researchers should estimate the expected distribution of scores when determining “good” vs. “bad” or default to the recommendations of the NINDS-CDE (69).

Working with patients, health care providers and researchers we should identify domains that are most relevant to improving health care delivery. Work to develop consensus for a set of core outcome domains for aSAH is currently under way (72). This international collaboration of patients, researchers and health care providers will determine a limited set of outcome domains across the four main categories in Figure 2, timepoints and the measurement instruments to create a core outcome set (COS). COS reduce research waste, improve consistency, and enable better comparison between studies.

## Conclusion

Selection of appropriate MI is a critical step in the design of robust clinical research. In this article we have characterized this process as considering three main components, what to measure, when to measure, and how to measure. Significant improvements have been made in this area with respect to standardization and the development of aSAH specific MI, but there remains a need for simplified aSAH specific functional measures. Efforts to reduce the potential mismatch between the outcomes selected by researchers and the users of the research (patients and health care providers) is crucial. The development of a COS in aSAH should help to address any mismatch and will be another significant step forward in improving research efficiency and generating more high-level research in this devastating condition.

## Author contributions

CA conceived the review and wrote the initial draft of the manuscript. AD and SE provided significant edits and assistance with restructuring the subsequent drafts. All authors read and approved the final version.

## Funding

This work was supported by the Ottawa Hospital Academic Medical Organization Innovation Fund Grant provided to improve outcome measurement in subarachnoid hemorrhage.

## Conflict of interest

The authors declare that the research was conducted in the absence of any commercial or financial relationships that could be construed as a potential conflict of interest.

## Publisher's note

All claims expressed in this article are solely those of the authors and do not necessarily represent those of their affiliated organizations, or those of the publisher, the editors and the reviewers. Any product that may be evaluated in this article, or claim that may be made by its manufacturer, is not guaranteed or endorsed by the publisher.
